# Malnutrition and Food Insecurity Might Pose a Double Burden for Older Adults

**DOI:** 10.3390/nu12082407

**Published:** 2020-08-11

**Authors:** Konstantinos Gkiouras, Stavros Cheristanidis, Theopoula D. Papailia, Maria G. Grammatikopoulou, Nikolaos Karamitsios, Dimitrios G. Goulis, Theodora Papamitsou

**Affiliations:** 1 Medical School, Faculty of Health Sciences, Aristotle University of Thessaloniki, GR-54124 Thessaloniki, Greece; t.papailia@gmail.com (T.D.P.); mariagram@auth.gr (M.G.G.); 2 Laboratory of Atmospheric Physics, Department of Physics, Aristotle University of Thessaloniki, University Campus, GR-54124 Thessaloniki, Greece; stavrcher@auth.gr; 3 School of Chemical Engineering, National Technical University of Athens, GR-15780 Athens, Greece; 4 Primary Health Care Center, GR-63080 Nea Kallikratia, Greece; karamitsiosnikos@yahoo.gr; 5 Unit of Reproductive Endocrinology, 1st Department of Obstetrics and Gynecology, Medical School, Faculty of Health Sciences, Aristotle University of Thessaloniki, GR-56429 Thessaloniki, Greece; dgg@auth.gr; 6 Laboratory of Histology and Embryology, Medical School, Faculty of Health Sciences, Aristotle University of Thessaloniki, GR-54124 Thessaloniki, Greece

**Keywords:** multimorbidity, health care use, health utilization, financial crisis, food availability, aging, chronic disease, malnutrition, food insecurity, food security

## Abstract

Although food insecurity has been associated with a disadvantageous socioeconomic status, especially in older adults, its association with comorbidities is less clear. The scope of the present cross-sectional study was to assess the prevalence of food insecurity among older adults and evaluate the association between food insecurity, malnutrition, chronic disease, multimorbidity and healthcare utilization. A total of 121 older adults (mean (standard deviation) age: 72.6 (8.1)) were recruited from a Primary Care Health Center from 10 August 2019 to 10 September 2019. Food insecurity and malnutrition status were assessed by the Household Food Insecurity Access Scale and Mini Nutritional Assessment tool, respectively. Recorded variables included financial, family data and comorbidities. The prevalence of food insecurity in the sample reached 50.4%, with men and older adults malnourished or at risk for malnutrition, exhibiting high risk for food insecurity. Multimorbidity, frequency of health care utilization and medication adherence were not associated with food insecurity, possibly due to the free health services and remunerated medications offered by the Greek government. However, male gender and malnutrition risk were significant predictors of food insecurity in the multiple logistic analyses. This study highlights the need for mainstreaming food insecurity assessment among older adults with comorbidities, especially those at risk for malnutrition.

## 1. Introduction

Food insecurity is a contemporary health issue among older adults [1,2,3], especially in Westernized countries that suffered the recent financial crisis [4,5]. Since food insecurity is dependent on household income, any health expenses [6,7] and, subsequently, diseases increasing medication costs and hospitalization needs, act as risk factors for food insecurity. Food-insecure individuals tend to make frequent use of the healthcare system [8,9,10,11,12], with a collateral increase in national healthcare expenditures [13]. In parallel, multimorbidity, a common problem during older age, has been shown to have a reciprocal association with food insecurity in the US, with either situation inflating the other [14,15,16,17].

Older adults in particular are prone to malnutrition for a variety of physiological, psychological and economic reasons [18,19,20]. Malnutrition is an often-under-recognized condition, despite the fact that it is associated with increased hospitalization and health care costs, as well as a greater disease burden [21,22,23]. According to the recent literature, the prevalence of free-living older adults at risk for malnutrition in Greece is high [4,5,19], as a possible epiphenomenon of the dire economic situation. Due to the sensitivity of both food insecurity and malnutrition to financial factors, it has been suggested that often, the two conditions coincide among older adults, augmenting the need for proper nutritional triage and prompt care [4,24].

It has been estimated that, in Greece, 69% of the older adult population endure some degree of food insecurity [4] as a result of the financial crisis austerity measures, including sharp income adjustments and pension reductions [25]. With the Greek Health System offering pro bono services to all pensioners through state Health Care Centers and Hospitals. In this context, the present cross-sectional study was designed to assess the prevalence of food insecurity among older adults visiting a Primary Care Health Center and evaluate the association between food insecurity, malnutrition, chronic disease, multimorbidity and healthcare utilization.

## 2. Materials and Methods

### 2.1. Population Recruitment

Older adults were recruited from the Primary Care Health Center at Nea Kallikratia, Northern Greece, from 10th August 2019 to 10th September of the same year. Participants were considered eligible when (1) they had an age exceeding 60 years, (2) could understand and communicate efficiently in the Greek language, (3) agreed to participate in the study by providing informed consent. Exclusion criteria included all those unwilling to participate.

### 2.2. Food Insecurity Assessment

Assessment of food insecurity was performed using the globally applied Household Food Insecurity Access Scale (HFIAS) [26]. The HFIAS consists of nine questions assessing uncertainty and anxiety about food access, quality and quantity of food consumption during the last four weeks. The score spans between 0–27, with higher scores indicating increased food insecurity [26]. Each question assesses a food insecurity domain with a yes/no answer and following a positive answer an increasing occurrence of this domain is explored (with possible answers being ‘rarely,’ ‘sometimes’ and ‘often’). The HFIAS consists of four categories; (1) food secure, (2) mild food insecure, (3) moderate food insecure and (4) severely food insecure. The categorization is based on equations which take into consideration both the rate of the answer and the order of the question. For instance, an individual can be categorized as food secure if they have answered positively (‘rarely’) only to the first question. The HFIAS has been previously used in the Greek population [4,27].

### 2.3. Malnutrition Assessment

Malnutrition status was assessed with the Greek version of the Mini Nutritional Assessment (MNA^®^) tool (Nestlé, Vevey, Switzerland) [28]. According to the MNA score, participants were stratified into three tiers, those who were considered well-nourished (MNA > 23.5), those at risk for malnutrition (17 < MNA ≤ 23.5) and the malnourished ones (MNA < 17).

### 2.4. Family Status, Lifestyle and Financial Data

For each participant, socio-economic data were collected, including educational level, marital status, number of children and cigarette smoking habits. Financial data, namely monthly income (€), having a loan, helping a family member financially and having an unemployed family member, were also recorded.

### 2.5. Anthropometric Assessment

Bodyweight and height of each participant were recorded in morning hours, at the Primary Care Health Center, by an experienced dietitian (T.P.), with the use of a digital scale (SECA 813, SECA Group, Hamburg, Germany) and a wall-mounted stadiometer (SECA 216, SECA Group, Hamburg, Germany), respectively. Body Mass Index (BMI) was calculated as body mass (kg), divided by height squared (m).

### 2.6. Comorbidities

For each participant, the Charlson Comorbidity Index (CCI) was calculated [29] based on the age and the number of known disease diagnoses. The index provides a score ranging from 0 to 37, based on various health issues, with a lower score being indicative of fewer comorbidities.

### 2.7. Ethical Approval

All subjects gave their informed consent for inclusion before they participated in the study. The study was conducted in accordance with the Declaration of Helsinki and the protocol was approved by the Primary Care Health Center director and the Bioethical Committee of Aristotle University of Thessaloniki’s Department of Medicine (project ID: 4.208, date of approval: 17 July 2019).

### 2.8. Statistical Analyses

Descriptive statistics were presented as frequencies and percentages (for qualitative variables) or as medians, with their first and third quartiles (for quantitative variables). To avoid sparse data in analyses, categorical variables were combined as follows—food-secure vs. all food-insecure categories (by combining mild, moderate and severely insecure participants), well-nourished vs. at malnutrition risk (at risk for malnutrition and malnourished grouped) and married vs. not-married (single/widower/divorced participants combined). Differences between food-secure and food-insecure participants were assessed with the Mann-Whitney U test (for quantitative variables) and the chi-square or Fisher’s exact test.

Univariable logistic regression was performed to identify the association between food insecurity (dependent variable) and each independent variable. For the multiple logistic (ML) examination of this association, a purposeful selection of the independent variables was applied as previously described [30,31]. First, variables associated with food insecurity at a significance level of *p* < 0.200 were selected and a ML model was constructed (ML model 1). ML model 1 included gender, marital status, paying off a loan, treatment adherence, monthly frequency of primary health care visits and malnutrition status. Subsequently, a second ML model (ML model 2) was constructed by including only those variables with a *p*-value < 0.05 in the ML model 1, namely gender and malnutrition status. As a sensitivity analysis, the models were re-constructed for examining the reciprocal relationship between malnutrition (dependent variable) and food insecurity (independent variable) by including the same covariates. Multicollinearity between independent variables was evaluated through the calculation of the variance inflation factor. The results of the logistic regressions are presented as odds ratios (OR) and their respective 95% confidence intervals (CI).

The level of significance was set at *α* = 0.05. All analyses were carried out on SPSS version 25.0 (IBM, SPSS Inc., Chicago, IL, USA).

## 3. Results

Data were collected for 150 individuals; however, 28 were excluded from the sample for being less than 60 years old and 1 was removed from the analyses due to missing data. Thus, the final sample consisted of 121 older adults. Table 1 describes the characteristics of participants according to food security status. Nearly half (49.6%) of the sample consisted of food-secure older adults and the remaining 50.4% demonstrated some degree of food insecurity. Men comprised 47.1% of the total sample; however, more men than women were diagnosed with food insecurity (*p* = 0.003), with male gender increasing the odds of being food insecure (OR: 3.08, 95% CI: 1.47–6.48). No differences were recorded between food insecurity and age, BMI or CCI.

Approximately all (95%) of the participants reported visiting the health care center due to a chronic health condition, with only a small proportion experiencing an acute health issue (5%). In parallel, 70.2% of the participants were at risk of developing malnutrition. In the univariable assessment, malnutrition risk status was associated with being food insecure (OR: 4.73, 95% CI: 1.98–11.30).

Following univariable logistic regressions, a ML analysis (Table 2) revealed an association with food-insecurity among participants of male gender (OR: 2.81, 95% CI: 1.25–6.32) and those at risk for malnutrition (OR: 4.46, 95% CI: 1.77–11.25). Family status, loan, treatment adherence, monthly frequency of public health care visits did not attenuate the association between malnutrition and food insecurity (Table 2). A second ML analysis of gender and malnutrition risk as independent variables did not reveal substantial changes in their associations with food insecurity (Table 2). Finally, a sensitivity analysis which examined the reciprocal relationship between malnutrition risk (dependent variable) and food insecurity revealed near identical results (Table S1).

## 4. Discussion

The present study revealed that a high proportion (50.4%) of older adults attending a Primary Care Health Center in Greece experienced food insecurity. Among food-insecure participants, being a male, malnourished or at risk for malnutrition was associated with higher odds of food insecurity. No differences were observed in the health care utilization frequency, disease diagnosis or multimorbidity between older adults of different food security status.

The prevalence of food insecurity in the present sample was relatively high, with approximately 50.4% of the participants exhibiting some degree of food insecurity. In an earlier study in Portugal, 23% of the older adults experienced food insecurity [32], whereas, during the year 2014, 10.2 million older Americans were reported to be food-insecure [33], with the prevalence increasing each year exponentially [34]. Increased prevalence of food insecurity has also been reported in previous research conducted in Greece, with food-insecure older adults exceeding 50% of the free-living older adults [4,27], as assessed with the HFIAS, indicating a similarly elevated food insecurity prevalence in the country and the older population in particular. Although a lack of relevant pre-recession data is apparent, proxy measures as food banks and food assistance indicate that the number of food-insecure older adults surged as an epiphenomenon of the financial crisis and the austerity measures taken by the government [4,35]. In the US, the prevalence of adult food insecurity inside the health care setting has been reported to range between 13.2–19.1% [12,13,36]; however, one possible explanation for the observed among-country difference lays to the fact that in Greece, health care provision is provided to all for free, whereas in the US only insured individuals make use of the health care resources. In parallel, even in the US, research in the private health care setting has revealed a much lower prevalence of food insecurity compared with the public sector [37]. Nevertheless, despite the importance of the issue, research on the prevalence of food insecurity within the healthcare setting appears limited and conducted mainly in the US.

During older age, elevated dietary intakes are required for many nutrients to achieve nutrient balance [38]. In parallel, an increased prevalence of age-related physiological changes and health issues affecting dietary intake is exhibited, including dental, swallowing or chewing problems, delayed satiety, sensory impairments, mobility limitations and an often-great dependency on others for feeding [39]. Due to slower movements, increased time is required to consume meals, often leading to a reduced volume in meal intake and less frequent snacking [40,41]. As a result, nutrient deficiencies and malnutrition are prevalent among older adults [38,41]. In the present sample, 70.2% of the participants were either malnourished or at risk for malnutrition, indicating that awareness about food insecurity among older adults in the primary care setting appears to be essential.

The findings verify that food insecurity among older individuals appears to be interrelated with malnutrition risk [42], with each situation propelling the other and both acting synergistically in hindering healthy aging. According to Lepore and Rochford [34], food insecurity is a primary risk factor for malnourishment. In parallel, both conditions share common drivers, including economic difficulties, deteriorating health and the existence of underlying disease [34]. On the other hand, according to the FAO, although longitudinal data are required to establish a causal relationship, food insecurity consists of an emerging, indisputable predictor of undernutrition [43]. Previous research in Greece has associated malnutrition and food insecurity among older adults [4], whereas, in the U.S., poor nutritional status has been reported among food-insecure older adults [44,45]. According to the U.S. Senate Special Committee on Ageing [46], individuals with food insecurity often exhibit “treat or eat” behavior, trading foods for other necessities, including medication or house expenses. It has been estimated that approximately 1/3 of Americans with a chronic condition exhibit this “treat or eat” behavior [47], inevitably leading to poor nutritional status and malnutrition. In the U.K., indirect food insecurity measures indicate that 1.4% of emergency food parcel recipients during the year 2017 were older adults [48]. In parallel, a great majority of lunch club attendants are older adults [49,50], with their dietary intake being significantly improved during lunch-club days [51].

According to the analysis, a greater risk for food insecurity was observed among older men, as compared to women. Overall, men have a reduced life expectancy compared to women [52]. In contrast, it has been suggested that women are physiologically more adaptive to famines and food shortages [53], with the greater fat deposition among females being a possible explanation for the latter. Nevertheless, the link between food insecurity and male sex in the present sample is unclear.

In most research conducted outside Greece, food insecurity has been associated with increased morbidity, medication non-adherence and frequency of health care usage [11,32,47]. On the other hand, chronic disease and, in particular, multimorbidity is associated with substantially increased health care costs [54,55,56]. For some populations, these costs cannot always be met, and, as a result, food insecurity is often prevalent among multimorbid older adults [10,14,15,16,32]. In the present sample, multimorbidity assessed with the CCI had no effect on food security status; however, it should be noted that in Greece, most medications are remunerated by the government, minimizing out-of-pocket costs of the citizens. As a result, no difference was observed in the CCI between different food security status participants. In contrast, in countries where medications are bought on the patient’s expense, substantial medication under-use has been reported among food-insecure patients [47,57], in a possible attempt to reduce health care costs and increase cash availability temporarily.

In parallel, given the aforementioned association between multimorbidity and food insecurity, US-based research has suggested that food-insecure individuals tend to make extended use of the health care services, irrespectively of their age [6,12]. Due to the differences in the two healthcare systems, this finding was not verified in the present sample. For most participants, visits to the Primary Care Health Center were pre-scheduled, based on the need for medication prescriptions and check-ups. Subsequently, no difference was noted in the use of health care services between food-secure and food-insecure older adults. Thus, despite the dire economic situation, the pro bono health services offered to all citizens in Greece and the remuneration of most medications [58] might reduce the health care and medication expenses of Greek older adults to a great extent, tampering down food insecurity in the country.

Bringing attention to policymakers, experts and stakeholders concerning food insecurity and its impact on older adults’ health are crucial [42], especially in the times of COVID-19, when, according to the FAO [59,60], food insecurity is expected to surge as a result of unemployment and decreased food availability. In parallel, raising awareness concerning food insecurity among health workers is also important in understanding possible medication non-adherence and compromised health. Nevertheless, the assessment of food insecurity in the health care setting is of pivotal importance. As a result, incorporation of routine screening in the standard practice has been suggested by the American Academy of Pediatrics [61], the American Diabetes Association [62] and the American Association of Retired Persons [63]. These recommendations reflect the increasing interest in understanding patients’ social determinants of health and providing patient-centered healthcare services [64,65].

Limitations of the present study include the relatively small sample recruited from the Primary Care Health Center and the cross-sectional design, which did not allow causal inferences. Nevertheless, the included population consisted of the total number of older adults attending the Center during sample recruitment, willing to participate in the study and assessed key associations among financial variables, comorbidities and food insecurity status.

## 5. Conclusions

In Greece, an alarmingly high proportion of older adults attending outpatient clinics experience food insecurity, often coinciding with malnutrition. Integration of food security screening in the standard practice is essential to understand the driving forces behind morbidity, deliver patient-oriented health care and offer solutions to reduce food insecurity in this population [66].

## Figures and Tables

**Table 1:** Sample’s characteristics with a univariable assessment of food-secure versus food-insecure participants ^d^.

Variables		Total Sample (*n = 121*)	Food-Secure (*n* = 60)	Food-Insecure (*n* = 61)	*p*-Value ^1^	OR ^2^	95% CI	*p*-Value
Demographics:	Men	57 (47.1%)	20 (33.3%)	37 (60.7%)	0.003	3.08	1.47–6.48	0.003
	Age (years)	72.0 (66.0–78.0)	72.5 (65.5–80.0)	72.0 (66.0–76.0)	0.498	0.98	0.94–1.03	0.377
	BMI (kg/m^2^)	29.0 (26.5–30.8)	28.8 (26.4–30.8)	29.1 (26.6–30.8)	0.693	1.03	0.94–1.12	0.583
	Smoking	34 (28.1%)	16 (26.7%)	18 (29.5%)	0.728	1.15	0.52–2.55	0.728
	≤10 cigarettes per day (vs. none)	14 (11.6%)	6 (10.0%)	8 (13.1%)	0.790	1.30	0.42–4.08	0.650
	>10 cigarettes per day (vs. none)	22 (18.2%)	12 (20%)	10 (16.4%)		0.81	0.32–2.09	0.668
	Not-married (single/widower/divorced)	26 (21.5%)	16 (26.7%)	10 (16.4%)	0.169	0.54	0.22–1.31	0.172
Financial data:	Paying off a loan	20 (16.5%)	6 (10.0%)	14 (23.0%)	0.055	2.68	0.95–7.53	0.061
	Having an unemployed family member	7 (5.8%)	4 (6.7%)	3 (4.9%)	0.717 ^F^	0.72	0.16–3.38	0.681
	Helping a family member financially	14 (11.6%)	9 (15.0%)	5 (8.2%)	0.242	0.51	0.16–1.61	0.249
	Monthly income (€/capita)	800 (600–1000)	800 (580–1000)	800 (600–1200)	0.192	1.00	0.999–1.001	0.638
Health data:	Often/Rare treatment adherence	27 (22.3%)	10 (16.7%)	17 (27.9%)	0.139	1.93	0.80–4.66	0.142
	Acute health problem was a reason for visiting the primary health care facility	6 (5.0%)	3 (5.0%)	3 (4.9%)	1.000 ^F^	1.02	0.20–5.25	0.983
	Primary health care visits frequency (>1/month)	8 (6.6%)	2 (3.3%)	6 (9.8%)	0.272 ^F^	3.16	0.61–16.35	0.169
	Carlson Comorbidity Index (CCI)	4 (3–5)	4 (3–5)	4 (3–5)	0.689	0.93	0.70–1.23	0.606
	Malnutrition risk	85 (70.2%)	33 (55.0%)	52 (85.2%)	<0.001	4.73	1.98–11.30	<0.001
Disease status:	Cardiovascular disease	2 (1.7%)	1 (1.7%)	1 (1.6%)	1.000 ^F^	0.98	0.06–16.09	0.983
	Hypertension	56 (46.3%)	29 (48.3%)	27 (44.3%)	0.653	0.85	0.42–1.74	0.653
	Renal disease	5 (4.1%)	2 (3.3%)	3 (4.9%)	1.000 ^F^	1.50	0.24–9.31	0.663
	Type 1 diabetes mellitus	15 (12.4%)	6 (10.0%)	9 (14.8%)	0.428	1.56	0.52–4.68	0.430
	Type 2 diabetes mellitus	47 (38.8%)	21 (35.0%)	26 (42.6%)	0.390	1.38	0.66–2.88	0.390
	Pulmonary disease	5 (4.1%)	2 (3.3%)	3 (4.9%)	1.000 ^F^	1.50	0.24–9.31	0.663

*BMI*, body mass index; *CI*, confidence intervals; *OR*, odds ratios. ^d^ Data are presented as frequencies (with percentages) for qualitative variables and as medians (with quartiles) for quantitative variables. ^1^ Differences between food-secure and insecure participants were assessed with chi-square or Fisher’s exact (denoted as ^F^) test for qualitative variables and Mann-Whitney U test for quantitative variables. ^2^ Univariable logistic regressions between food-secure (reference category) versus food-insecure participants.

**Table 2:** Assessment of food-secure (*n* = 60) versus food-insecure (*n* = 61) participants.

	ML1			ML2		
Variable	OR	95% CI	*p*-Value	OR	95% CI	*p*-Value
Men	2.81	1.25–6.32	0.013	3.19	1.44–7.03	0.004
Not-married (single/widower/divorced)	0.65	0.24–1.74	0.392	-	-	-
Paying off a loan	2.16	0.68–6.85	0.193	-	-	-
Often/Rare Treatment adherence	1.68	0.62–4.52	0.307	-	-	-
Visits in primary health care > 1/month	2.46	0.36–16.91	0.361	-	-	-
Malnutrition risk	4.46	1.77–11.25	0.002	4.87	1.97–12.06	0.001

*CI*, confidence intervals; *ML1*, multiple logistic model 1, including variables found to be significant in the univariable assessment, at *p* < 0.200 (Table 1); *ML2*, multiple logistic model 2, including variables found to be significant in the ML1, at *p* < 0.05.; *OR*, odds ratio.

## Supplementary Materials

The following is available online at https://www.mdpi.com/2072-6643/12/8/2407/s1, Table S1: Sensitivity analysis: multiple logistic assessment of well-nourished (*n* = 36) versus malnutrition risk (*n* = 85) participants.
