# Supplementary material for: Malnutrition and Food Insecurity Might Pose a Double Burden for Older Adults
Source: Nutrients. 2020 Aug 11;12(8):2407. doi: 10.3390/nu12082407 (PMC7468760; doi:10.3390/nu12082407)
Supplement: Supplementary file 1 [file nutrients-12-02407-s001.pdf]

nutrients MDPI

Supplementary Material:

Malnutrition and food insecurity pose a double burden for older adults

Konstantinos Gkiouras, Stavros Cheristanidis, Theopoula D. Papailia, Maria G. Grammatikopoulou, Nikolaos Karamitsios, Dimitrios G. Goulis and Theodora Papamitsou

Table S1. Sensitivity analysis: multiple logistic assessment of well-nourished (*n*=36) versus malnutrition risk (*n*=85) participants.

	ML1			ML2		
Variable	OR	95% CI	p-value	OR	95% CI	p-value
Men	0.87	0.36–2.09	0.748	0.90	0.38–2.14	0.811
Not-married (single/widower/divorced)	0.71	0.26–1.89	0.487	-	-	-
Paying off a loan	1.30	0.37–4.60	0.680	-	-	-
Often/Rare Treatment adherence	1.05	0.36–3.02	0.935	-	-	-
Visits in primary health care > 1/month	2.33	0.25–21.72	0.458	-	-	-
Food insecurity	4.45	1.77–11.18	0.001	4.87	1.97–12.06	0.001

CI, confidence intervals; *ML1*, multiple logistic model 1; *ML2*, multiple logistic model 2; *OR*, odds ratio. ML1 and ML2 performed as a sensitivity analysis examining the reciprocal relationship between malnutrition risk (dependent variable) and food insecurity (independent variable) while including the same set of covariates presented in Table 2.
